# Molecular mechanisms for the prevention and promoting the recovery from ischemic stroke by nutraceutical laminarin: A comparative transcriptomic approach

**DOI:** 10.3389/fnut.2022.999426

**Published:** 2022-08-24

**Authors:** Jiefeng Luo, Dingzhi Chen, Biyun Qin, Deyan Kong

**Affiliations:** Department of Neurology, The Second Affiliated Hospital of Guangxi Medical University, Nanning, China

**Keywords:** laminarin, ischemic stroke, inflammation, comparative transcriptomics, molecular mechanisms, nutraceutical, blood vessel development

## Abstract

Stroke is the second leading cause of death and a major cause of disability worldwide. Ischemic stroke caused by atherosclerosis accounts for approximately 87% of all stroke cases. Ischemic stroke is a preventable disease; therefore, a better understanding of the molecular mechanisms underlying its pathogenesis and recovery processes could provide therapeutic targets for drug development and reduce the associated mortality rate. Laminarin, a polysaccharide, is a nutraceutical that can be found in brown algae. Accumulating evidence suggests that laminarin could reduce the detrimental effects of neuroinflammation on brain damage after stroke. However, the molecular mechanism underlying its beneficial effects remains largely unknown. In the present study, we used a middle cerebral artery occlusion (MCAO) rat model and applied comparative transcriptomics to investigate the molecular targets and pathways involved in the beneficial effects of laminarin on ischemic stroke. Our results show the involvement of laminarin targets in biological processes related to blood circulation, oxygen supply, and anti-inflammatory responses in the normal brain. More importantly, laminarin treatment attenuated brain damage and neurodeficits caused by ischemic stroke. These beneficial effects are controlled by biological processes related to blood vessel development and brain cell death through the regulation of canonical pathways. Our study, for the first time, delineated the molecular mechanisms underlying the beneficial effects of laminarin on ischemic stroke prevention and recovery and provides novel therapeutic targets for drug development against ischemic stroke.

## Introduction

Ischemic stroke, referred to brain ischemia and cerebral ischemia, accounts for approximately 87% of all stroke cases (1). According to data from the Centers for Disease Control and Prevention, over 795,000 people in the United States are diagnosed with stroke every year,^1^ resulting in nearly $53 billion stroke-related costs in 2018 (1). Stroke is the second leading cause of death and a major cause of disability worldwide, but 80% of strokes are preventable (2). A better understanding of the molecular mechanisms underlying the pathogenesis and recovery processes of ischemic stroke would provide novel therapeutic targets for drug development against ischemic stroke.

The use of nutraceutical compounds is considered an alternative therapeutic approach for ischemic stroke and for promoting recovery (3). Laminarin is a polysaccharide of glucose that is commonly found in brown algae, such as *Laminaria sp.* Laminarin is a macromolecule with anti-tumor, anti-inflammatory, immunostimulatory, antioxidant, and anticoagulant activities, and was recently found to induce angiogenesis (4). Few studies have shown that laminarin exhibits neuroprotective effects against forebrain ischemia injury by attenuating oxidative stress and neuroinflammation (5). However, the molecular mechanisms underlying these protective effects remain unclear. The middle cerebral artery occlusion (MCAO) model is commonly used to study the mechanism of ischemic stroke, in which a surgical filament is inserted into the internal carotid artery, resulting in the blockage of blood flow and subsequent brain infarction in the MCA area (6–8). The MCAO model has been used to investigate the neuroprotective effects of compounds against ischemic stroke as well (9, 10).

In the present study, we used the MCAO rat model and performed comparative transcriptome sequencing, followed by systematic bioinformatic analysis, including Gene Ontology (GO) and Ingenuity Pathway Analysis (IPA), to delineate the molecular mechanisms underlying the beneficial effects of laminarin in preventing ischemic stroke and promoting the recovery from ischemic stroke through the promotion of blood vessel functions and anti-inflammation. More importantly, our report is the first to delineate the involvement of growth factors, transporters, transmembrane receptors, G-protein-coupled receptors, enzymes, kinases, and transcription regulators in the recovery of ischemic stroke mediated by laminarin.

## Materials and methods

### Animal maintenance and middle cerebral artery occlusion model establishment

Male Sprague-Dawley rats (240 ± 20 g, specific pathogen-free, 9 weeks old) were obtained from the Central Animal Facility of Guangxi Medical University (Nanning, China). The animals were housed in clean cages under standard conditions and light and dark cycles (12 h:12 h; temperature: 25°C) with free access to food and water. All animal studies were conducted according to the approved protocols and guidelines of the Institutional Animal Ethical Care Committee of the Guangxi Medical University Experimental Animal Center. The cerebral ischemia model was established as previously described (11). Briefly, the rats were anesthetized with chloral hydrate (10%, 3 mL/kg), the inner and outer muscles of the sternocleidomastoid muscles were separated to expose and isolate the right common, external, and internal carotid arteries. The occlusion was performed by inserting a monofilament (approximately 2 cm) from the external carotid artery to the middle cerebral artery, avoiding the pterygopalatine artery. After the monofilament was inserted, the common carotid artery was ligated to complete the occlusion and induce ipsilateral ischemia. After 2 h of ischemia, the monofilament was gently pulled out, and ligation of the common carotid artery was relieved to cause reperfusion. The wound was disinfected with iodine and sutured. The MCAO model was confirmed using three monitors. Laser Doppler flowmetry (LDF)-guided fiber insertion was used to monitor ipsilateral blood flow, modified neurological severity score (mNSS) was used to evaluate overall neurological function, and triphenyl tetrazolium chloride staining was used to evaluate infarct size.

### Experimental design and laminarin treatment

The rats were randomly divided into four groups as follows: (1) the negative control group: no surgery; (2) the laminarin treatment group: no surgery, but underwent intraperitoneal injection of laminarin (dissolved in normal saline) at 10 mg/kg per day for 7 days; and (3) the MCAO group: the rats were subjected to 2 h ischemia followed by 24 h reperfusion, and then were intraperitoneally injected with the same amount of normal saline as the laminarin treatment group daily for 7 days. (4) MCAO + laminarin treatment group: rats were intraperitoneally injected with laminarin (dissolved in normal saline) at 10 mg/kg per day for 7 days after MCAO.

### Neurological deficit evaluation

The modified neurological severity score (mNSS) was used to evaluate the neurobehavioral outcomes every day after MCAO for seven days, as previously reported (11). There are four tests in the scoring system: motor, sensory, balance, and reflex tests. Scores from all tests were summed, where 0 represented no deficit and 18 represented maximal deficit.

### Infarct volume assessment

At day 7 after reperfusion and treatment, the rats were euthanized under general anesthesia by cardiac puncture. Brains were immediately removed and cut into five serial 2 mm-thick coronal slices. A 2% solution of 2,3,5-triphenyltetrazolium chloride (TTC) was used to assess the infarct zone (white zone). Image-J^®^ (image-processing software) was used by an independent observer blinded to the group status to measure the ischemic area (the unstained areas) and calculate the infarct volume in each rat brain (*n* = 3 per group). Infarct area was calculated as the area of the non-ischemic hemisphere minus the non-infarcted area of the ischemic hemisphere. Infarct volume = infarct area × thickness (2 mm). The percentage of cerebral infarction was calculated using the following formula: The percentage of cerebral infarction = infarct volume/the volume of the non-ischemic hemisphere × 100%.

### Comparative transcriptome sequencing and bioinformatic analysis

The cerebral cortex ischemic penumbra was isolated for transcriptome sequencing after the rats were sacrificed (five rats per condition). The cerebral ischemic core area of the MCAO rats was white, and the brain tissue around the ischemic penumbra was regarded as the ischemic core area. Total RNA was extracted using TRIzol reagent, following the manufacturer’s instructions, as previously described (12). High-quality RNA samples with an RNA integrity number > 7.0 were used to construct the sequencing library as previously described (12). Paired-end sequencing (PE150) of 2 × 150 bp was performed using a DNBSEQ sequencer as the customer service provided by the Beijing Genomics Institute. The clean reads (after the removal of low-quality reads) were aligned to the rat reference genome (rn6) using the HISAT2 (version:hisat2-2.0.4^2^) package, and StringTie and ballgown^3^ were used to determine the gene expression. Comparative transcriptomic analysis was performed using DESeq2 software. Genes with |log2 fold change| ≥ 0.5 and false discovery rate < 0.05 were considered differentially expressed genes (DEGs). The DEGs were then subjected to GO enrichment analysis and IPA to determine the beneficial roles of laminarin in ischemic stroke.

### Statistical analysis

All values in this study were presented as the mean ± SEM of replicates. Data were analyzed using GraphPad Prism 8 (GraphPad, United States). Independent sample *t*-tests were performed to assess whether there was a significant difference between control and treatment groups. A *p*-value of < 0.05 was considered significant.

## Results

### Laminarin improved the recovery of neurobehavioral outcome in middle cerebral artery occlusion model

**FIGURE 1:**
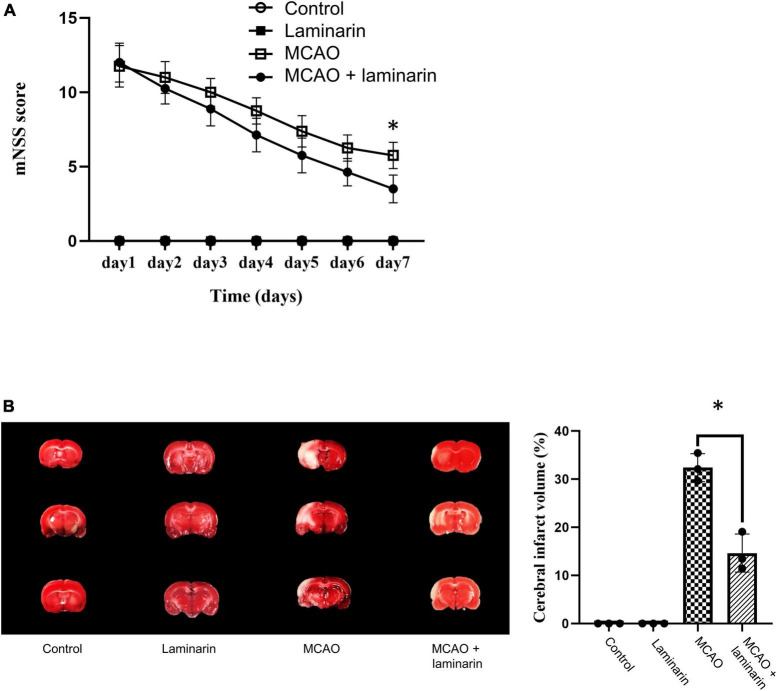
Laminarin treatment reduced the neuro-deficits in MCAO model. **(A)** The modified neurologic severity score was used to evaluate the neuro-clinicopathological deficits. The score was higher in the MCAO model and this induction was significantly attenuated after 7 days of laminarin treatment. **(B)** The infarct volume (white zone) of brain was higher in MCAO model, which was significantly reduced after 7 days of laminarin treatment. *N* = 8 per condition. * Represented *p*-value < 0.05 and statistically significant.

The mNSS score was used to evaluate the neurobehavioral outcomes in the MCAO model after laminarin treatment. Our results showed a significant reduction in the score after seven days of laminarin treatment in the MCAO model (Figure 1A). In addition, the induced infarct volume, which reflected the clinicopathological deficits in the brain caused by ischemic stroke, was attenuated by laminarin treatment (Figure 1B). Taken together, our data suggest that laminarin treatment could promote the recovery of neurodeficits in the MCAO model.

### Beneficial role of laminarin in the maintenance of brain functions

**FIGURE 2:**
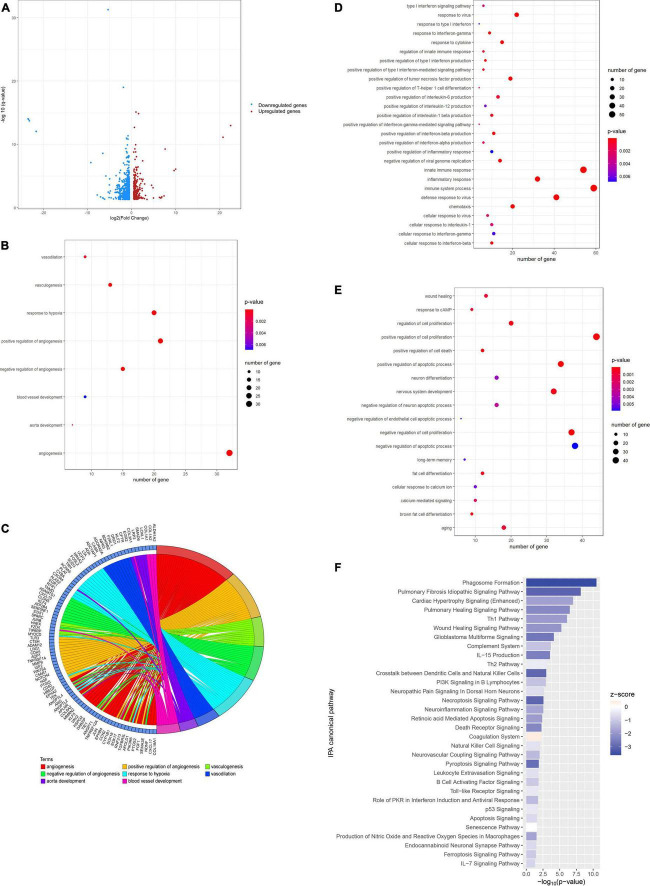
Laminarin regulated blood vessel development and promoted the brain function. **(A)** Volcano plot showing the differential gene expression in normal brain caused by laminarin treatment. Blue dots represent downregulated genes and red dots represent upregulated genes. **(B)** Circos plot showing the involvement of laminarin-targeted gene cluster in blood vessel-related processes. Gene ontology enrichment analysis highlighted the involvement of laminarin in biological processes related to **(C)** blood circulation and oxygen supply, **(D)** immune and inflammatory responses, and **(E)** brain functions. The size of the bubble represents the number of genes and the color of bubble represents the significance of the biological process. **(F)** Canonical Ingenuity Pathway Analysis (IPA) showed the inhibition of immune response and cell death processes in the normal brain. Negative *z*-score represents the inhibition of pathways.

To understand the molecular mechanism underlying the beneficial effect of laminarin in the prevention of ischemic stroke, a comparative transcriptomic analysis was performed. When we compared the transcriptomic profiles of the control and laminarin groups, 957 differentially expressed genes (DEGs), including 275 upregulated and 682 downregulated genes, were identified (Figure 2A). GO enrichment analysis of these DEGs highlighted the effect of laminarin on biological processes related to blood circulation and oxygen supply, including angiogenesis, vasculogenesis, vasodilation, aorta development, blood vessel development, and response to hypoxia (Figure 2B), which were regulated by a cluster of genes (Figure 2C). In addition, laminarin regulated many processes related to immune and inflammatory responses, such as the production of interleukin, regulation of T-helper 1 cell differentiation, response to cytokines, and response to interferon-gamma (Figure 2D). Cell proliferation and apoptosis were also regulated (Figure 2E). More importantly, laminarin was found to play a role in many brain-related functions, including nervous system development, brown fat cell differentiation, neuron differentiation, regulation of neuronal apoptotic processes, aging, calcium-mediated signaling, long-term memory, response to cAMP, and cellular response to calcium ions (Figure 2E). The canonical pathway analysis using IPA further highlighted the inactivation of immune and inflammatory responses (Figure 2F), which was reflected by the negative *Z*-score values. In addition, cell death-related signaling (including necroptosis signaling pathway, death receptor signaling, retinoic acid-mediated apoptosis signaling, ferroptosis signaling pathway, apoptosis signaling, and p53 signaling) and brain-related disorders (including pyroptosis signaling pathway, glioblastoma multiforme signaling, neurovascular coupling signaling pathway, endocannabinoid neuronal synapse pathway, and neuropathic pain) in the brain were inhibited by laminarin (Figure 2F). Additionally, pathways related to the circulatory system such as pulmonary fibrosis idiopathic signaling, pulmonary healing signaling, wound healing signaling, cardiac hypertrophy signaling, and coagulation system were regulated by laminarin (Figure 2F). Taken together, our data suggest the potential protective role of laminarin in brain function against ischemic stroke through the regulation of inflammatory response and blood vessels.

### Recovery roles of laminarin in middle cerebral artery occlusion model

**FIGURE 3:**
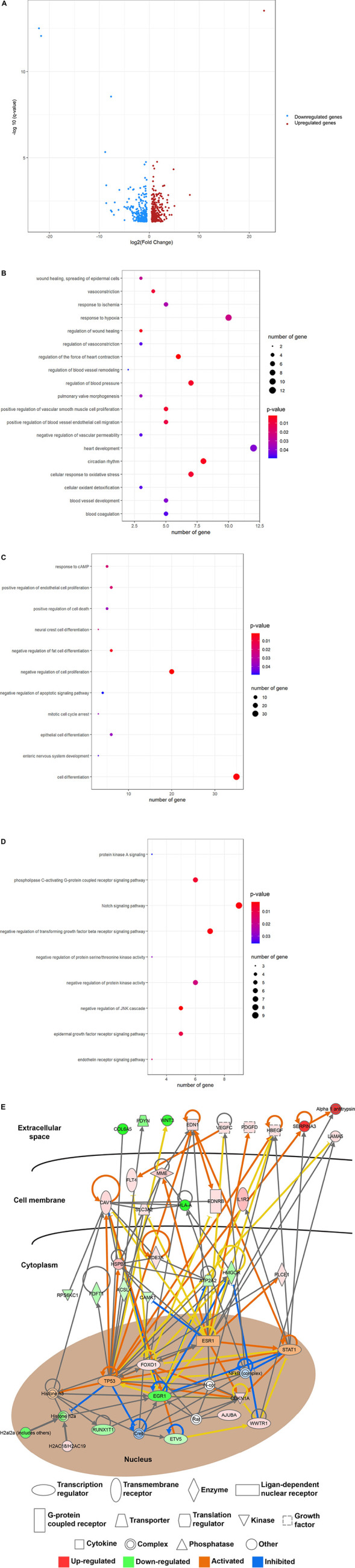
Beneficial effect of laminarin in MCAO model. **(A)** Volcano plot showing the differential gene expression in the brain of MCAO model caused by laminarin treatment. Blue dots represent downregulated genes and red dots represent upregulated genes. Gene ontology enrichment analysis highlighted the involvement of laminarin in biological processes related to **(B)** development of blood vessel, **(C)** brain functions, and **(D)** cell signaling pathways. The size of the bubble represents the number of genes and the color of the bubble represents the significance of the biological process. **(E)** Gene networking of IPA demonstrated the involvement of growth factors, transporters, transmembrane receptors, G-protein coupled receptors, enzymes, kinases, and transcription regulators in the recovery roles of laminarin in ischemic stroke. Red figures represent the upregulated genes and green figures represent downregulated genes.

We then determined the recovery roles of laminarin after ischemic stroke and compared the transcriptomic profile between the MCAO and MACO + laminarin groups. Comparative transcriptomic analysis identified 555 DEGs, including 289 upregulated and 266 downregulated genes (Figure 3A). In line with the recovery effect of laminarin in the MCAO model, the results of functional annotation suggested the involvement of these genes in biological processes related to the development of blood vessels, including vasoconstriction, regulation of blood vessel remodeling, regulation of blood vessel endothelial cell migration, response to ischemia, and regulation of vascular permeability (Figure 3B). In addition, the biological processes related to brain cell function and development, such as regulation of fat cell differentiation, response to cAMP, neural crest cell differentiation, and enteric nervous system development, were highlighted, suggesting the positive role of laminarin in promoting brain functions in MCAO rats (Figure 3C). All these processes were mediated by the regulation of different cell signaling pathways, such as the JNK cascade, Notch signaling, transforming growth factor beta receptor signaling, phospholipase C-activating G-protein coupled receptor signaling, epidermal growth factor receptor signaling, endothelin receptor signaling, regulation of protein kinase activity, regulation of protein serine/threonine kinase activity, and protein kinase A signaling (Figure 3D). The gene network construction of canonical pathway analysis in IPA showed the involvement of growth factors, transporters, transmembrane receptors, G-protein coupled receptors, enzymes, kinases, and transcription regulators in the beneficial effects of laminarin against ischemic stroke (Figure 3E). Taken together, our data suggest that laminarin plays a recovery role in ischemic stroke by promoting the blood supply and brain function.

## Discussion

In this study, we aimed to investigate the effects of laminarin on ischemic stroke and the molecular mechanisms regulated by it, using an MCAO rat model. Our results showed that treatment with laminarin could attenuate the neuro-deficits in MCAO model. This finding was concordant with a previous report that laminarin could act as a dectin-1 antagonist to improve neurological function after ischemic stroke (13). Other than the inhibition of dectin-1, other possible molecular targets and pathways controlled by laminarin are still unknown. Therefore, we applied comparative transcriptomic analysis and bioinformatics analysis to completely delineate the mechanisms underlying the roles of laminarin in preventing ischemic stroke and the beneficial recovery outcome. In the bioinformatics analysis, we mainly focused on the biological processes and functions related to the pathogenesis and recovery of ischemic stroke, including inflammatory responses, blood vessel development, and oxygen supply. Inflammation plays a critical role in the pathogenesis and secondary cell death in ischemic stroke (14). Hence, the progressive inflammation that occurs after stroke can serve as a therapeutic target to prevent secondary brain injury (15).

This study was divided into two parts. First, we aimed to determine the effect of laminarin on preventing ischemic stroke and its protective effect on normal brain function. The results of comparative transcriptomic analysis highlighted that laminarin mediates many biological processes related to inflammatory and immune responses, suggesting its pivotal role in anti-neuroinflammation. More importantly, our results suggest that laminarin can control biological processes related to vasculogenesis, vasodilation, and angiogenesis through the regulation of a cluster of genes. Some of these targets have already been shown to play a role in ischemic stroke. For instance, calcium-sensing receptor (CASR) has been reported to be associated with the pathophysiology of stroke (16), and it can act as a target for ischemic neuroprotection (17). Adenosine A2A receptor (ADORA2A), which belongs to the family of adenosine receptors that are most abundant in the brain, has been reported to contribute to neuroinflammation and synaptic and neuronal damage (18) and is considered a biomarker of brain diseases. A study using adenosine A2A receptor-null (Adora2a-/-) mice demonstrated its role in the coronary reactive hyperemic response and vascular contraction (19). Another study using Adora2a-/- mice showed that inactivation of endothelial ADORA2A protected mice from cerebral ischemia-induced brain injury and improved post-stroke outcomes through anti-inflammatory effects and blockade of NLRP3 inflammasome activity (20).

In addition to the reported candidates, our results also highlight some novel therapeutic targets to improve the quality of blood vessels for preventing ischemic stroke, such as adenylate cyclase activating polypeptide 1 (ADCYAP1), natriuretic peptide receptor-C (NPR3), and bradykinin receptor B2 (BDKRB2). ADCYAP1 is known as the pituitary adenylate cyclase-activating peptide (PACAP), which is a multifunctional neuropeptide (21). PACAP has been reported to be actively transported across the blood-brain barrier and plays a role in headache disorders, such as migraine (22). A study on PACAP-deficient mice demonstrated its physiological role in the regulation of vascular tone (23). Another mammalian study in rats also suggested a vasorelaxant effect of PACAP in basilar arteries through the activation of nitrergic neurons (24). Another novel target, NPR3, is expressed in endocardial endothelial cells and the entire endocardium (25, 26). Many studies have demonstrated the role of NPR3 in vascular function. A genetic lineage tracing study showed that the involvement of NPR3 in the endocardium contributed to a minority of coronary vessels in the free walls of the embryonic heart (26). Another genome-wide association study on human primary vascular smooth muscle cells and endothelial cells from different individuals identified that genetic variants at the NPR3 locus are associated with elevated blood pressure (27). NPR3 was found to be downregulated in human aortic sections subjected to high blood pressure from coarctation of the aorta (28), suggesting that NPR3 could be a therapeutic target for vascular disease prevention. In addition to NPR3, BDKRB2 has also been found to be involved in endothelial function and blood vessel constriction (29, 30), thus determining cardiovascular health and regenerative arteriogenesis. A study on senescence-accelerated OXYS rats suggested that BDKRB2 plays a significant role in the development of cerebrovascular dysfunction (31). A human genetic study of BDKRB2 polymorphisms showed an association between BDKRB2 and peripheral vasoconstriction (32). This is supported by another human genotype study in which the BDKRB2 BE1 polymorphism influences bradykinin type 2 receptor-mediated vasodilation during angiotensin-converting enzyme inhibition (33), suggesting a protective role of BDKRB2 in the vascular response. Thus, all targets of laminarin could be used for drug development to prevent ischemic stroke through the improvement of blood vessel development and relaxation.

In the second part of the study, we investigated the beneficial role of laminarin in recovery outcome after ischemic stroke. GO enrichment showed that laminarin plays an important role in blood vessel development and wound healing. These findings support previous reports that laminarin stimulates tissue regeneration and angiogenesis, and increases blood vessel density, leading to the promotion of wound healing (34, 35). Canonical IPA further delineated the molecular network of this important feature of laminarin. The results of gene networking showed that laminarin stimulated growth factors (VEGFC, PDGFD, and HBEGF), which are known to play multiple roles in restoring vascular function. Vascular endothelial growth factor C (VEGFC) is a secreted glycoprotein that induces angiogenesis (36). VEGFC promotes the growth of vessels and revascularization in the central nervous system (37). A photothrombotic ring stroke rat model demonstrated that VEGFC promotes early angiogenesis, leading to spontaneous reperfusion after stroke (38). In addition, VEGFC was found to improve the survival of neural stem cells (39) and stimulate the proliferation of both early and late oligodendrocyte progenitors in the subventricular zone after neonatal hypoxia-ischemia (40). Other growth factors, including platelet-derived growth factor-D (PDGFD), are potent transforming and angiogenic growth factors (41). A PDGFD transgenic mouse study showed that PDGFD could induce blood vessel maturation during angiogenesis (42). More importantly, a case-control study demonstrated an association between PDGFD gene polymorphisms and ischemic stroke (43). Heparin-binding EGF-like growth factor (HBEGF), a hypoxia-inducible neuroprotective protein, was reported to play a neuroprotective role by stimulating the proliferation of neuronal precursor cells during ischemia and reperfusion injury (44). Sugiura and colleagues showed that HBEGF enhanced neurogenesis and angiogenesis after focal cerebral ischemia in a MCAO rat model (45). A similar study showed that post-ischemic administration of HBEGF reduced infarct size and induced neurogenesis after focal cerebral ischemia, further supporting its beneficial role (46). Therefore, laminarin-stimulated growth factors may have beneficial effects on the recovery of ischemic stroke.

In addition to growth factors, our results also showed that laminarin could target membrane proteins, such as laminin subunit alpha-5 (LAMA5) and caveolin-1 (CAV1). Both have been reported to play a protective role in blood–brain barrier (BBB) integrity (47, 48). An endothelium-specific lama5-knockout mouse study showed the contribution of endothelial LAMA5 to BBB maintenance and its beneficial role in intracerebral hemorrhage (47). In addition, LAMA5 promotes proliferation and angiogenesis of human umbilical vein endothelial cells (49). Cav1 is an integrated protein located in the caveolar membrane, which can promote nerve regeneration and angiogenesis *via* the CAV1/VEGF pathway (48). In addition, a CAV1-knockout mouse study demonstrated the role of endogenous CAV1 in promoting neovascularization, astrogliosis, and scar formation after ischemia (50). In terms of its immunosuppressive role, laminarin stimulated interleukin 1 receptor type 2 (IL1R2) and inhibited human leukocyte antigen A (HLAA) in an ischemic stroke model. Although no studies have correlated the level of IL1R2 with stroke, a study on cardiomyocytes showed that IL-1R2 protected the heart from ischemia and reperfusion injury (51). HLA-A is a major type of MHC class I transmembrane protein that is critical for T-cell controlled immune response (52). A correlation study suggested an association between human leukocyte antigen alleles and risk of stroke in the Iranian population (53).

In conclusion, our results provide evidence that laminarin treatment could have beneficial effects on the prevention of ischemic stroke and promote recovery outcomes after stroke. These beneficial effects are mediated by a cluster of genes that are involved in blood vessel development, vascular function, and immunosuppression. However, all these results were only at the transcription level; therefore, a follow-up study investigating the expression of these proteins at the post-transcription level could warrant the findings from our study. Moreover, preclinical human studies are needed to verify these findings before laminarin can be used clinically.

## Data availability statement

The datasets presented in this study can be found in online repositories. The names of the repository/repositories and accession number(s) can be found below: https://www.ncbi.nlm.nih.gov/, PRJNA861444.

## Ethics statement

The animal study was reviewed and approved by the Guidelines of the Care and Use of Laboratory Animals of the National Institutes of Health and approved by the Ethics and Animal Subject Committee of Guangxi Medical University.

## Author contributions

JL, DC, and DK contributed to the conception and design of the study, wrote the first draft of the manuscript, and wrote sections of the manuscript. BQ organized the database. JL and BQ performed the statistical analysis. All authors contributed to the manuscript revision, read, and approved the submitted version.

## Footnotes

1
https://www.cdc.gov/stroke

2
https://daehwankimlab.github.io/hisat2/

3
http://www.bioconductor.org/packages/release/bioc/html/ballgown.html
